# Effects of Automated Versus Conventional Ventilation on Quality of Oxygenation—A Substudy of a Randomized Crossover Clinical Trial

**DOI:** 10.3390/jcm14010041

**Published:** 2024-12-25

**Authors:** Michela Botta, David M. P. van Meenen, Tobias D. van Leijsen, Jitske R. Rogmans, Stephanie S. List, Pim L. J. van der Heiden, Janneke Horn, Frederique Paulus, Marcus J. Schultz, Laura A. Buiteman-Kruizinga

**Affiliations:** 1 Department of Intensive Care, Amsterdam UMC, University of Amsterdam, 1105 AZ Amsterdam, The Netherlands; bottamichela@gmail.com (M.B.); l.kruizinga@rdgg.nl (L.A.B.-K.); 2 Department of Intensive Care, Dijklander Hospital, 1624 NP Hoorn, The Netherlands; 3 Department of Intensive Care, Reinier de Graaf Hospital, 2625 AD Delft, The Netherlands; 4 Amsterdam Neurosciences, Amsterdam UMC, University of Amsterdam, 1105 AZ Amsterdam, The Netherlands; 5 Urban Vitality, Centre of Expertise, Faculty of Health, Amsterdam University of Applied Sciences, 1102 ST Amsterdam, The Netherlands; 6 Mahidol–Oxford Tropical Medicine Research Unit (MORU), Mahidol University, Bangkok 10400, Thailand; 7 Nuffield Department of Medicine, University of Oxford, Oxford OX3 7BN, UK; 8 Department of Anesthesia, General Intensive Care and Pain Management, Medical University Wien, 1090 Vienna, Austria

**Keywords:** intensive care, invasive ventilation, automated ventilation, closed-loop ventilation, oxygenation, fraction of oxygen, positive end-expiratory pressure, ventilator alarms, ventilator settings, manual interventions

## Abstract

**Background/Objectives**: Attaining adequate oxygenation in critically ill patients undergoing invasive ventilation necessitates intense monitoring through pulse oximetry (SpO_2_) and frequent manual adjustments of ventilator settings like the fraction of inspired oxygen (FiO_2_) and the level of positive end-expiratory pressure (PEEP). Our aim was to compare the quality of oxygenation with the use of automated ventilation provided by INTELLiVENT–Adaptive Support Ventilation (ASV) vs. ventilation that is not automated, i.e., conventional pressure-controlled or pressure support ventilation. **Methods**: A substudy within a randomized crossover clinical trial in critically ill patients under invasive ventilation. The primary endpoint was the percentage of breaths in an optimal oxygenation zone, defined by predetermined levels of SpO_2_, FiO_2_, and PEEP. Secondary endpoints were the percentage of breaths in acceptable or critical oxygenation zones, the percentage of time spent in optimal, acceptable, and critical oxygenation zones, the number of manual interventions at the ventilator, and the number and duration of ventilator alarms related to oxygenation. **Results**: Of the 96 patients included in the parent study, 53 were eligible for this current subanalysis. Among them, 31 patients were randomized to start with automated ventilation, while 22 patients began with conventional ventilation. No significant differences were found in the percentage of breaths within the optimal zone between the two ventilation modes (median percentage of breaths during automated ventilation 19.4 [0.1–99.9]% vs. 25.3 [0.0–100.0]%; *p* = 0.963). Similarly, there were no differences in the percentage of breaths within the acceptable and critical zones, nor in the time spent in the three predefined oxygenation zones. Although the number of manual interventions was lower with automated ventilation, the number and duration of ventilator alarms were fewer with conventional ventilation. **Conclusions**: The quality of oxygenation with automated ventilation is not different from that with conventional ventilation. However, while automated ventilation comes with fewer manual interventions at the ventilator, it also comes with more ventilator alarms.

## 1. Introduction

Ensuring adequate oxygenation levels while avoiding the risks of hyperoxia or hypoxia is crucial for critically ill patients undergoing invasive ventilation [1,2,3,4]. Hyperoxia can lead to oxidative stress and tissue damage, while hypoxia may result in organ dysfunction and failure. Achieving this balance requires frequent and precise manual adjustments of ventilator settings, such as the fraction of inspired oxygen (FiO_2_) and positive end-expiratory pressure (PEEP). These adjustments are based on the patient’s rapidly changing physiological needs and require intensive monitoring by skilled intensive care unit (ICU) nursing staff, adding to their workload.

The use of automated ventilation modes is becoming increasingly appealing in the care of critically ill patients as they have the potential to reduce the workload associated with invasive ventilation [5,6]. One of these modes, named INTELLiVENT–Adaptive Support Ventilation (ASV), employs sophisticated algorithms capable of selecting and adjusting ventilator settings such as the tidal volume (V_T_) and respiratory rate (RR), as well as FiO_2_ and PEEP. The algorithms are based on both inputs from the patient, i.e., end-tidal carbon dioxide (etCO_2_) and pulse oximetry (SpO_2_) sensors, and information inserted into the machine by the healthcare provider, including gender and height, target ranges for etCO_2_ and SpO_2_, and limits for maximum airway pressure and PEEP. INTELLiVENT–ASV changes the ventilator settings based on algorithms that target a low work and force of breathing [7,8] and this affects settings related to oxygenation, i.e., FiO_2_ and PEEP. It is uncertain whether this mode of automated ventilation performs as well as conventional ventilation with regard to oxygenation and whether it reduces the number of interventions and alarms of the ventilator.

We performed a substudy within a randomized crossover clinical trial in critically ill patients under invasive ventilation with the aim of comparing the quality of oxygenation with the use of automated ventilation provided by INTELLiVENT–ASV vs. non-automated ventilation using conventional pressure-controlled or pressure support ventilation. Additionally, we assessed the impact of the automated ventilation mode on the number of manual interventions at the ventilator, as well as the number and duration of ventilator alarms related to oxygenation. The primary hypothesis was that using the automated ventilation mode would result in patients spending more time within the optimal oxygenation range.

## 2. Materials and Methods

### 2.1. Design and Patients

This is a substudy of ‘Effect of automated versus conventional ventilation on mechanical power of ventilation—a randomized crossover clinical trial’ (INTELLiPOWER). INTELLiPOWER is an international, multicenter, randomized crossover clinical trial, conducted in hospitals in the Netherlands and Switzerland. The study protocol of INTELLiPOWER was approved by the Institutional Review Board (IRB) of the Amsterdam UMC, location ‘AMC’, Amsterdam, the Netherlands (2020_317#B2021122), and the Cantonal Ethics Commission Zurich (Swissethics) (2023–D0012). The study was registered at clinicaltrials.gov (study identifier NCT04827927). Prior to inclusion in the study, written consent was secured from the legal representative of each participant. The current substudy did not require additional approval or informed consent. The primary outcome of the parent study INTELLiPOWER was to investigate the mechanical power of ventilation in passive and active invasively ventilated patients, between automated ventilation and conventional ventilation; the recently published results of the INTELLiPOWER study [9] showed that the automated ventilation mode INTELLiVENT–ASV reduced mechanical power in passive patients. The current substudy focuses on oxygenation and its parameters as primary endpoints, i.e., FiO_2_, PEEP, and SpO_2_, and changes in ventilation settings and alarms as secondary endpoints.

Patients were eligible for inclusion in the parent study if they met the following criteria: (1) were 18 years of age or older; (2) were anticipated to require invasive ventilation for a minimum of 24 h; (3) could be randomized promptly, but no later than 48 h after the initiation of invasive ventilation in the ICU; and (4) were ventilated using an ICU ventilator capable of delivering the automated ventilation mode under investigation, namely, INTELLiVENT–ASV. Exclusion criteria for INTELLiPOWER were receiving invasive ventilation through a tracheostomy cannula, a body mass index above 40 kg/m^2^, and any contraindications to the use of the automated ventilation mode to be tested. This substudy did not use other inclusion and exclusion criteria. However, patients could only participate in this substudy if breath-by-breath ventilation data were collected from the ventilator using a specific mass storage device.

### 2.2. Study Interventions

Participants were randomized in a 1:1 ratio to either automated ventilation or non-automated conventional ventilation, which could be pressure-controlled or pressure support ventilation, for a duration of 3 h. They then crossed over to the alternate ventilation mode. A 30 min washout period was applied between the two crossover phases. Randomization was performed using random block sizes of 4 or 6 patients and was stratified by center, utilizing a secure, password-protected, web-based randomization system (SSL-encrypted website, Castor Electronic Data Capture, Amsterdam, the Netherlands). Due to the nature of the intervention, blinding was not possible.

The predecessor of INTELLiVENT–ASV, known as ASV, is a ventilation mode designed to adapt to a patient’s respiratory mechanics, automatically adjusting V_T_ and RR as needed. This approach simplifies ventilatory management by tailoring settings to a patient’s condition. Building on this, INTELLiVENT–ASV uses advanced algorithms and real-time monitoring to enhance ventilation optimization. It adjusts PEEP and FiO_2_ using dedicated controllers while finetuning V_T_ and RR through a minute ventilation controller. With INTELLiVENT–ASV, ventilation and oxygenation targets are defined as ranges for etCO_2_ and SpO_2_. The ventilator continuously adjusts V_T_, RR, PEEP, and FiO_2_ on a breath-by-breath basis to meet these targets.

All doctors and nurses involved in the study were qualified to use the automated ventilation mode, with extensive prior experience. Consecutive patients were randomized to receive automated or conventional ventilation; this was performed as soon as possible after the initiation of ventilation, but always within 48 h after the start of ventilation in the ICU. During the crossover phases of the study, similar targets for etCO_2_ and SpO_2_ were employed. The depth of sedation was maintained at the same level, and patients were not exposed to additional activities such as routine care, physiotherapy, or airway interventions unless deemed absolutely essential during the two crossover phases of the study.

During automated ventilation, the target ranges for SpO_2_ and etCO_2_ were determined by the attending ICU physician, ICU nurse, or ventilation practitioner, using the same goals as those set prior to randomization. The ventilator’s minute volume, PEEP, and FiO_2_ controllers were activated, enabling the software to adjust parameters such as V_T_, RR, PEEP, and FiO_2_ based on the algorithms of the ventilator mode. The PEEP lower limit was set to 5 cm H_2_O and the upper PEEP limit ranged from 10 to 15 cm H_2_O, depending on the patient’s lung mechanics or clinical status and in line with the local ventilation protocol.

During conventional pressure-controlled or pressure support modes, the attending ICU doctor, ICU nurse, or ventilation practitioner adjusted the settings of the ventilation mode based on the same targets for SpO_2_ and etCO_2_ established before randomization. Lung-protective settings were recommended as per the local ventilation protocol, including the use of low V_T_ (6–8 mL/kg predicted body weight [PBW]), low maximum airway pressure (Pmax 30 cm H_2_O), and driving pressure below 15 cm H_2_O. PEEP was set based on the lower PEEP/FiO_2_ table [10], with the lowest allowed PEEP being 5 cm H_2_O. FiO_2_ was adjusted to maintain SpO_2_ within the desired range.

### 2.3. Data Collected

The following data were collected and stored—demographic data, including gender, age, height, weight, and chronic comorbidities; the Acute Physiology and Chronic Health Evaluation (APACHE) IV, reason for ICU admission and mechanical ventilation, and date and time of hospital and ICU admission and start of ventilation. Ventilation settings and parameters before randomization were also collected, including the mode of ventilation, humidification system in use, V_T_, RR, minute volume, Pmax, PEEP, set inspiratory pressure or set pressure support where applicable, flow, inspiratory time, FiO_2_, SpO_2_, etCO_2_, and arterial blood gas analysis data.

Breath-by-breath ventilation data were collected during the whole study period of interest (~6–1/2 h) on a specific mass storage device. Data were then converted into a comma-separated value (CSV) file and stored in a restricted access folder. Data collection included the date and time of each breath, oxygenation settings and parameters, i.e., FiO_2_, PEEP, SpO_2_, status of the automation controllers (automated, manual, or frozen), and oxygenation-related ventilator alarms.

### 2.4. Zone of Oxygenation

Breaths were classified into three predefined zones of oxygenation based on the measurements of peripheral oxygen saturation by pulse oximetry, PEEP, and FiO_2_, as shown in Supplementary Table S1. In detail, an optimal, acceptable, or critical SpO_2_ zone value was assigned to each breath based on SpO_2_ values suggested in the literature [11,12,13]. Afterward, each PEEP/FiO_2_ combination was scored based on an adapted low-PEEP/high-FiO_2_ table. Every breath was then classified into optimal, acceptable, or critical based on the two previous values.

### 2.5. Outcomes

The primary endpoint was the percentage of breaths in the predefined optimal oxygenation zone, calculated for each subject with the following equation:

percentage of breaths in zone = (number of breaths in zone/total number of breaths) × 100(1)

Secondary endpoints were the percentages of breaths in the predefined acceptable and critical oxygenation zones and the percentage of time spent in the optimal, acceptable, and critical oxygenation zones. Other secondary endpoints included the number of manual interventions at the ventilator and the number and duration of ventilator alarms related to oxygenation.

### 2.6. Statistical Analysis

We did not perform a formal sample size calculation. Instead, the number of patients in whom breath-by-breath data were collected and stored served as the sample size.

Data are expressed in numbers and percentages for categorical variables and medians with interquartile ranges or means with standard deviation for continuous variables. For the primary endpoint, data are presented as medians with interquartile ranges and means with standard deviation; a Wilcoxon-signed rank test was used to compare the percentage of breaths in the optimal zone in the two ventilation modes. The same test was used to compare the percentage of breaths in the acceptable and critical zone. The number and duration of alarms and the number of manual interventions were compared using a Wilcoxon-signed rank test.

Oxygenation variables data are visualized in cumulative distribution plots, wherein the median values of SpO_2_, PEEP, and FiO_2_ are shown. Percentages of breaths in the predefined zones are shown in bar plots, wherein the mean percentages of breath in the optimal, acceptable, and critical zones per arm are shown for the oxygenation zones and for SpO_2_ and PEEP/FiO_2_ zones separately.

No assumptions were made for missing data. All analyses were conducted in R Studio v. 4.2.1 (R Foundation, Vienna, Austria). The significance level was set at 0.05.

## 3. Results

**Figure 1:**
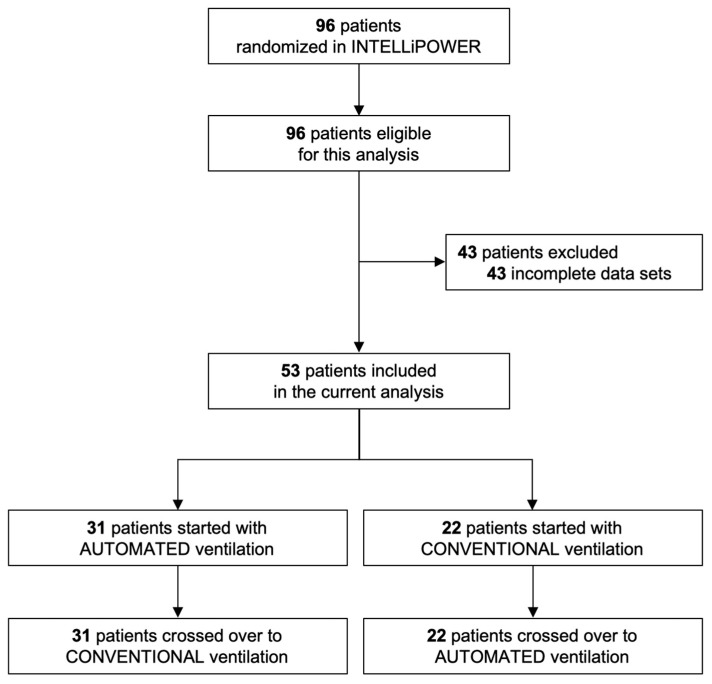
CONSORT flow diagram.

**Figure 2:**
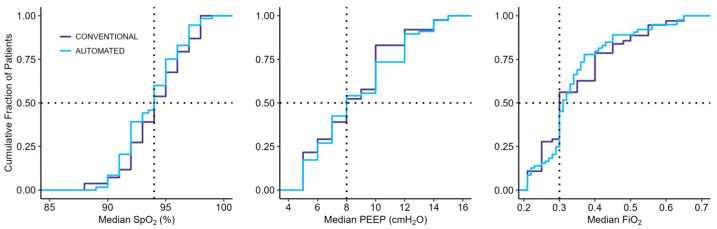
Cumulative distribution plots for SpO_2_, PEEP, and FiO_2_. The plots show the median variables with automated ventilation (light blue) and conventional ventilation (purple). Horizontal dotted lines represent 50% of the patients and vertical dotted lines represent median values for the conventional group. Abbreviations: SpO_2_, pulse oximetry; PEEP, positive end-expiratory pressure; FiO_2_, fraction of inspired oxygen.

Of the 96 patients enrolled in the main study, 46 were excluded because of missing or incomplete breath-by-breath data. Consequently, 53 patients were included in the current subanalysis, of which 31 were randomized to start with automated ventilation and 22 with conventional ventilation (Figure 1). Across all patents, a total of 356,598 breaths were collected: 183,014 breaths during the conventional ventilation crossover phase and 173,584 during the automated ventilation phase. Most patients were male, with the primary cause for ICU admission being a medical condition, and the most common indication for invasive ventilation was respiratory failure (Table 1). The majority of patients were randomized within 24 h of the initiation of invasive ventilation in the ICU. The ventilation parameters SpO_2_, PEEP, and FiO_2_ were not different between the conventional modes, as shown in Figure 2. Other ventilation variables and parameters were not different either, except for RR, which was higher during conventional ventilation (Figure 2 and Supplementary Table S2).

### 3.1. Primary Endpoint

**Figure 3:**
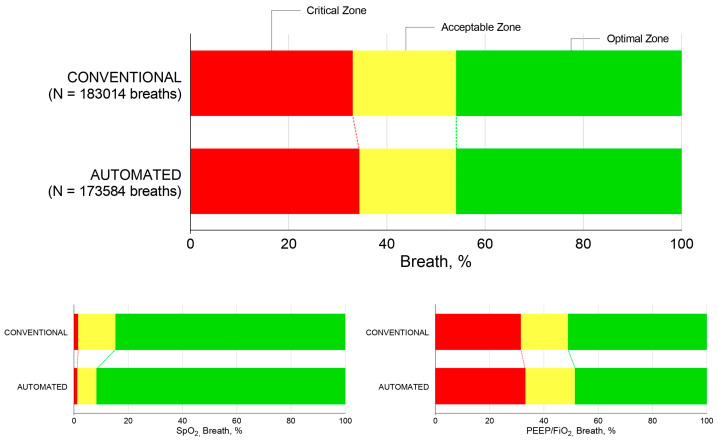
Proportions of breaths in predefined zones of oxygenation. Percentages of breaths in the predefined zones are shown in bar plots, wherein the mean percentages of breath in the optimal, acceptable, and critical zones per crossover phase are shown for the oxygenation zones and for SpO_2_ and PEEP/FiO_2_ zones separately.

There was no difference in the percentage of breaths in the optimal zone between automated and conventional ventilation (median percentage of breaths in the optimal zone with automated ventilation, 19.4 [0.1–99.9]% vs. 25.3 [0.0–100.0]% and mean percentage of breaths in the optimal zone with automated ventilation, 45.9 (45.4) vs. 45.9 (47.2); *p* = 0.963) (Table 2 and Figure 3).

### 3.2. Secondary Endpoints

The percentage of breaths within the acceptable and critical zones and the percentages of time spent in the three predefined oxygenation zones were not different between the two modes (Figure 2 and Table 2). This was also true for the percentages of time in the three predefined zones for SpO_2_ and PEEP/FiO_2_.

Compared to conventional ventilation, the number of manual interventions at the ventilator with automated ventilation was significantly lower but the number and duration of ventilator alarms related to oxygenation were significantly higher (Table 3).

## 4. Discussion

The findings of this substudy of a randomized crossover clinical trial comparing automated ventilation with conventional ventilation in invasively ventilated critically ill patients can be summarized as follows: (1) automated ventilation performs equally well as conventional ventilation in terms of oxygenation quality; and (2) ventilator settings related to oxygenation, i.e., FiO_2_ and PEEP, do not differ significantly between the two modes of ventilation. With automated ventilation, however, (3) the number of manual interventions at the ventilator is lower, (4) but the number and duration of ventilator alarms related to oxygenation are higher.

This study is the first randomized clinical trial to evaluate the impact of automated ventilation using INTELLiVENT–ASV on various aspects of oxygenation, as previous studies comparing automated ventilation with conventional ventilation exclusively focused on the effects on SpO_2_ and did not consider PEEP and FiO_2_. In two randomized studies involving patients after cardiac surgery, the primary endpoint was the time spent within optimal, acceptable, and critical zones of ventilation based on V_T_, the maximum airway pressure or plateau pressure, etCO_2_, and SpO_2_ [14,15]. In a study of COVID-19 ARDS patients, the primary endpoint was protective ventilation defined by V_T_, driving pressure, peak pressure, mechanical power, and SpO_2_ [16]. Another study involving critically ill patients expected to need ventilation for two days or more had an endpoint focused on the time spent with SpO_2_ values in optimal and non-optimal zones [17]. Additionally, in a mixed ICU cohort study, one of the endpoints was the time spent with SpO_2_ values within an acceptable range [18]. A systematic review showed that hyperoxemia occurred less often during INTELLiVENT–ASV [19], and in another systematic review, FiO_2_ was reduced by INTELLiVENT–ASV in diverse patient categories [19]. Unlike our study, in all but one of these studies, automated ventilation was found to be superior to conventional ventilation. The reasons for this discrepancy can only be speculated upon. One reason could be that the timespan of our study was much shorter, with data recorded for approximately 3 h during each mode of ventilation. Another reason could be that the patients included in our study had to be in a ventilatory stable situation, meaning that oxygenation did not exhibit significant fluctuations.

To the best of our knowledge, we are not aware of other studies that determined the effects of automated ventilation on the number of interventions at the ventilator related to oxygenation. Previous studies have assessed the total number of manual interventions at the ventilator related to various aspects of ventilation [5,14,17,20]. Our findings reveal that the number of manual adjustments at the ventilator related to oxygenation is significantly lower when using automated ventilation, aligning with the outcomes of similar studies. These prior studies also consistently demonstrated a reduction in the need for manual interventions with the implementation of automated ventilation. This decreased need for manual adjustments can be interpreted as a meaningful reduction in the workload for healthcare professionals, potentially allowing them to focus on other critical aspects of patient care. In resource-rich healthcare systems where ventilation standards are already high, the greatest benefit of automation may be in relieving the pressure on ICU staff rather than significantly improving patient outcomes. Automated ventilation like INTELLiVENT–ASV could help ease the workload of ICU nursing and medical staff by responding instantly to patients’ changing needs [21,22,23]. This is especially relevant in light of the growing challenges posed by ICU nursing staff shortages [24]. The importance of such systems became particularly evident during the COVID-19 pandemic, where a surge in patients requiring invasive mechanical ventilation placed significant strain on healthcare resources [25]. However, the workload related to mechanical ventilation encompasses more than just manual adjustments and alarm management [19]. Measuring staff workload is challenging, and to date, no studies have specifically examined ICU staff workload in relation to ventilation. While studies have consistently reported reductions in manual interventions, it is unclear whether this translates into an actual decrease in workload. This uncertainty is particularly relevant during specific phases of mechanical ventilation. This substudy focused on a relatively short period of mechanical ventilation, while the median duration of ventilation for this population was much longer (a median of 4 days, IQR 3–8 days in the INTELLiPOWER cohort). As such, even small differences in ventilator settings and alarms observed over a short period may have a cumulative and more significant impact over a longer duration. These findings, although from a small sample, suggest potential benefits that merit further investigation in larger studies with extended observation periods.

Our results also show that the number of alarms and their duration increases with automated ventilation. Earlier studies did not report data regarding the number and duration of alarms during INTELLiVENT–ASV, although recent studies comparing automated vs. conventional oxygen controllers during HFNO in both adult and pediatric populations showed a similar or lower number of alarms during automated oxygen control [26,27]. While alarms are of vital importance for patient safety, their continuous presence can negatively impact patients’ comfort and sleep hygiene. Additionally, the constant disruption to workflow and the need for manual interventions at the ventilator may increase the workload. Therefore, the correct setting of alarm limits is of utmost importance. In our study, it is important to note that the staff involved was trained and experienced in the use of the automated mode, so the higher number of alarms cannot be attributed to inexperience in alarm setting or improper use of the technology. This might be due to the fact that some alarms are not present in the conventional mode, e.g., when the FiO_2_ reaches a certain high threshold, or the alarm the machine gives when the oxygenation controller goes off because of various reasons. A higher number and duration of alarms is therefore to be expected, but regular checking of the correct alarm setting might prevent an overload. Our results, showing fewer manual interventions but a higher number and duration of alarms, suggest a complex clinical picture. While reduced manual interventions imply a lower workload for healthcare providers, the increase in alarms might indicate an increase in workload. This contradiction makes it difficult to determine the overall clinical benefit and highlights the need for further refinement of ventilator management.

Our study has the following strengths. We used a crossover design, wherein each patient serves as their own control, thereby enhancing the statistical power of the analysis. We conducted the study in ICUs of academic and non-academic hospitals across two countries, which increases the generalizability of the findings. The ICU nurses and doctors involved were all experienced in using the tested automated ventilation mode. Additionally, we gathered granular ventilation data, allowing breath-by-breath scoring and analysis. The automated collection of ventilator data also allowed a robust collection of manual interventions at the ventilator and of ventilator alarms related to oxygenation. Finally, we strictly adhered to the study protocol and the statistical analysis plan throughout the entire study and the analysis.

Our study does have certain limitations. The two crossover phases of the study were relatively short in duration, which raises the possibility that the effects of automated ventilation may differ when applied over a more extended time period. Additionally, the majority of patients included in the study were stable, both hemodynamically and respiratory, potentially limiting the applicability of our findings to unstable patients. Another aspect to consider is that patients were enrolled early after initiating ventilation, and the effects of the tested ventilation mode might vary in later phases of treatment. Due to the nature of the intervention, it was not possible to blind the study. Nonetheless, we strictly adhered to the study analysis plan, with all analyses performed by investigators who were blinded to the assigned ventilation mode. Finally, another limitation of this study is its focus on ICU settings, where the intensity and complexity of care are significantly higher than in general wards. As a result, the findings related to workload reduction and ventilator management may not directly translate to less acute environments. The unique demands of ICU care, including the higher frequency of ventilator adjustments and alarms, limit the generalizability of these results to other hospital settings where such interventions are less frequent.

## 5. Conclusions

Automated ventilation provides oxygenation of a similar standard as conventional ventilation. While it reduces the need for manual interventions at the ventilator, it is accompanied by an increase in the frequency and duration of ventilator alarms concerning oxygenation. Future studies are needed to determine if automated ventilation reduces the workload of ICU nursing staff.

## Figures and Tables

**Table 1:** Patient demographics, baseline, and ventilation characteristics.

	N = 53
Gender, male	40 (76)
Age, years	65 [56–73]
Height, cm	174 [167–180]
Weight, kg	80 [72–90]
Body mass index, kg/m^2^	26 [23–29]
APACHE IV score	79 [58–98]
**Comorbidities**	
cardiovascular disease	25 (47.2)
COPD	6 (11.3)
neurological condition	9 (17.0)
**Reason for ICU admission, no (%)**	
elective surgery	1 (1.9)
emergency surgery	6 (11.3)
medical condition	46 (86.8)
**Reason for mechanical ventilation, no (%)**	
respiratory failure	35 (66.0)
decreased consciousness	8 (15.1)
cardiac arrest	7 (13.2)
postoperative ventilation	3 (5.7)
Other	6 (11.3)
**Mode of ventilation**	
INTELLiVENT–ASV	37 (69.8)
pressure controlled	14 (26.4)
pressure support	2 (3.8)
PEEP, cmH_2_O	8 [6–10]
FiO_2_, %	31 [27–40]
SpO_2_, %	96 [92–97]
**Arterial blood gas analysis results**	
arterial pH	7.4 [7.3–7.4]
PaO_2_, kPa	9.8 [9.1–12.1]
PaCO_2_, kPa	5.4 [4.7–6.1]
bicarbonate, mmol	24 [20–27]
SaO_2_, %	94 [93–97]
PaO_2_/FiO_2_ ratio	243 [171–324]

Data are presented as numbers and percentages or medians and interquartile ranges. Abbreviations: APACHE, Acute Physiology and Chronic Health Evaluation; COPD, chronic obstructive pulmonary disease; ICU, intensive care unit; ASV, adaptive support ventilation; PEEP, positive end-expiratory pressure; FiO_2_, fraction of inspired oxygen; SpO_2_, pulse oximetry; PaO_2_, partial pressure of oxygen; PaCO_2_, partial pressure of carbon dioxide; SaO_2_, arterial oxygen saturation.

**Table 2:** Percentage of breaths in the predefined oxygenation zones.

	Automated Ventilation	Conventional Ventilation	*p*
Oxygenation zones			
Breaths in the optimal zone, %	19.4 [0.1–99.9] 45.9 (45.4)	25.3 [0.0–100.0] 45.9 (47.2)	0.963
Breaths in the acceptable zone, %	3.1 [0.0–32.9] 19.7 (28.2)	0.0 [0.0–36.2] 21.1 (34.0)	0.764
Breaths in the critical zone, %	3.5 [0.0–80.1] 34.4 (41.4)	0.4 [0.0–85.0] 33.1 (43.7)	0.772
Time in the optimal zone, %	19.4 [0.1–99.9] 45.9 (45.5)	24.9 [0.0–100.0] 46.1 (47.3)	0.776
Time in the acceptable zone, %	3.2 [0.0–31.7] 19.7 (28.4)	0.0 [0.0–36.6] 20.9 (33.9)	0.833
Time in the critical zone, %	3.6 [0.0–79.9] 34.3 (41.4)	0.3 [0.0–83.6] 33.0 (43.7)	0.984
**SpO_2_ zones**			
Breaths in the optimal zone, %	98.0 [89.1–100.0] 91.7 (13.6)	99.8 [80.3–100.0] 84.7 (27.0)	0.332
Breaths in the acceptable zone, %	2.0 [0.0–7.6] 7.1 (11.5)	0.2 [0.0–19.5] 13.8 (24.2)	0.339
Breaths in the critical zone, %	0.0 [0.0–0.0] 1.6 (6.5)	0.0 [0.0–0.1] 1.7 (6.5)	0.230
**PEEP/FiO_2_ zones**			
Breaths in the optimal zone, %	34.2 [3.5–100.0] 48.6 (44.5)	54.4 [0.0–100.0] 51.1 (48.4)	0.624
Breaths in the acceptable zone, %	2.1 [0.0–31.8] 18.3 (27.6)	0.0 [0.0–18.2] 17.4 (33.5)	0.460
Breaths in the critical zone, %	1.9 [0.0–80.1] 33.2 (41.2)	0.0 [0.0–84.8] 31.5 (44.3)	0.851

Data are presented as median [IQR] and means (SD). Abbreviations: SpO_2_, pulse oximetry; PEEP, positive end-expiratory pressure; FiO_2_, fraction of inspired oxygen; IQR, interquartile range; SD, standard deviation.

**Table 3:** Changes in ventilator settings and ventilator alarms related to oxygenation.

	Automated Ventilation	Conventional Ventilation	*p*
Changes in ventilator settings			
Automated changes in PEEP or FiO_2_, number per patient	32.0 [16.0–44.0] 33.9 (25.8)	N.A.	<0.001
Manual changes in PEEP or FiO_2_, number per patient	0.0 [0.0–2.0] 1.3 (2.4)	2.0 [0.0–3.0] 2.1 (2.3)	0.036
**Ventilator alarms**			
Ventilator alarms, number per patient	2.0 [1.0–8.0] 6.9 (11.4)	0.0 [0.0–2.0] 1.4 (2.4)	<0.001
Cumulative duration of alarms, minutes per patient	1.2 [0.1–6.8] 6.6 (10.6)	0.0 [0.0–2.1] 2.2 (4.9)	0.001

Data are presented as medians [IQR] and means (SD). Abbreviations: PEEP, positive end-expiratory pressure; FiO_2_, fraction of inspired oxygen; IQR, interquartile range; SD, standard deviation.

## Data Availability

The data used for this analysis are available from the corresponding author upon reasonable request including a statistical analysis plan.

## Supplementary Materials

The following supporting information can be downloaded at: https://www.mdpi.com/article/10.3390/jcm14010041/s1, Table S1: Predefined zones of oxygenation; Table S2: Ventilation and oxygenation parameters.
