# Supplementary material for: Effects of Automated Versus Conventional Ventilation on Quality of Oxygenation—A Substudy of a Randomized Crossover Clinical Trial
Source: J Clin Med. 2024 Dec 25;14(1):41. doi: 10.3390/jcm14010041 (PMC11721315; doi:10.3390/jcm14010041)
Supplement: Supplementary file 1 [file jcm-14-00041-s001.zip › jcm-3322655-supplementary.pdf]

Online Supplement to:

Effects of Automated *versus* Conventional Ventilation on Quality of Oxygenation – a substudy of a randomized cross–over clinical trial

Michela Botta[1], David M.P. van Meenen[1], Tobias D. van Leijsen[1], Jitske R. Rogmans[1], Stephanie S. List[2], Pim L.J. van der Heiden[3], Janneke Horn[1,4], Frederique Paulus[1,5], Marcus J. Schultz[1,6,7,8], and Laura A. Buiteman–Kruizinga[1,3] for the INTELLiPOWER*–investigators

*INTELLiPOWER, 'Effect of Automated versus Conventional Ventilation on Mechanical Power of Ventilation'

Amsterdam UMC, location University of Amsterdam, Amsterdam, the Netherlands
[1]Department of Intensive Care; [4]Amsterdam Neurosciences

Dijklander Hospital, location Hoorn, Hoorn, the Netherlands
[2]Department of Intensive Care

Reinier de Graaf Hospital, Delft, the Netherlands
[3]Department of Intensive Care

Amsterdam University of Applied Sciences, Faculty of Health, Amsterdam, the Netherlands
[5]Urban Vitality, Centre of Expertise

Mahidol University, Bangkok, Thailand
[6]Mahidol–Oxford Tropical Medicine Research Unit (MORU)

University of Oxford, Oxford, United Kingdom
[7]Nuffield Department of Medicine

Medical University Wien, Vienna, Austria
[8]Department of Anesthesia, General Intensive Care and Pain Management

Table of Contents

Table S1. Predefined zones of oxygenation page 3

Table S2. Ventilation and oxygenation parameters page 4

Table S1. Predefined zones of oxygenation.

	optimal	acceptable	critical
SpO_2	90–94% *or*<br>≥ 95% and FiO_2 < 40%	88–89% *or*<br>95–97% and FiO_2 ≥ 40%<br>≥ 98% and FiO_2 40–60%	<88% *or*<br>≥ 98% and FiO_2 ≥ 60%
PEEP*	according to low PEEP/high FiO_2 table ± 2 cmH_2O	according to low PEEP/high FiO_2 table ± 4 cmH_2O	according to low PEEP/high FiO_2 table ± 5 cmH_2O or more
Definition	if both present:<br>optimal zone	if not in the optimal zone *and* no critical is present:<br>acceptable zone	if one present:<br>critical zone

*optimal, acceptable and critical PEEP based on low PEEP/high FiO_2 table

FiO_2, fraction	0.3	0.4	0.5	0.6	0.7	0.8	0.9	1
optimal PEEP, cm H_2O	3–7	3–7	6–10	8–12	8–12	12–16	12–16	16–20
acceptable PEEP, cm H_2O	1–2 *or* 8–9	1–2 *or* 8–9	4–5 *or* 11–12	6–7 *or* 13–14	6–7 *or* 13–14	10–11 *or* 17–18	10–11 *or* 17–18	14–15 *or* 21–22
critical PEEP, cm H_2O	0 *or* ≥ 10	0 *or* ≥ 10	≤ 3 *or* ≥ 13	≤ 5 *or* ≥ 15	≤ 5 *or* ≥ 15	≤ 9 *or* ≥ 19	≤ 9 *or* ≥ 19	≤ 13 *or* ≥ 23

Abbreviations: SpO_2, pulse oximetry; PEEP, positive end–expiratory pressure; FiO_2, fraction of inspired oxygen.

Table S2. Ventilation and oxygenation parameters

	started with automated ventilation (N = 31)	started with conventional ventilation (N = 22)	*P*
Ventilation parameters			
V_T, mL	466 [404 – 561]	468 [432 – 535]	0.501
V_T, mL/kg PBW	6.7 [5.9 – 7.9]	7.0 [6.2 – 8.3]	0.777
RR, breaths/minute	18 [15 – 23]	19 [16 – 23]	0.028
Pmax, cmH_2O	21 [17 – 24]	21 [17 – 24]	0.944
PEEP, cmH_2O	8 [6 – 11]	8 [6 – 10]	0.262
FiO_2, %	32 [27 – 38]	30 [25 – 40]	0.996
SpO_2, %	94 [92 – 96]	94 [92 – 96]	0.479
$etCO_2$, kPa	4.9 [4.5 – 5.3]	4.8 [4.3 – 5.2]	0.167
Arterial blood gas analysis results			
pH	7.4 [7.4 – 7.4]	7.4 [7.3 – 7.4]	0.217
PaO_2, kPa	10.5 [9.1 – 11.6]	9.9 [9.1 – 11.9]	0.933
$PaCO_2$, kPa	5.5 [4.8 – 5.9]	5.3 [4.9 – 6.0]	0.714

Data are presented as medians and interquartile ranges.

Abbreviations: V_T, tidal volume; PBW, predicted body weight; RR, respiratory rate; Pmax, maximum airway pressure; PEEP, positive end–expiratory pressure; FiO_2, fraction of inspired oxygen; SpO_2, pulse oximetry; $etCO_2$, end–tidal carbon dioxide; PaO_2, partial pressure of oxygen; $PaCO_2$, partial pressure of carbon dioxide.
